# Perceived Carer Skills and Psychological Distress in Fathers and Mothers of Adults with Anorexia Nervosa: A Longitudinal Study

**DOI:** 10.1192/j.eurpsy.2026.12035

**Published:** 2026-07-31

**Authors:** M. N. Akkese, J. Keeler, J. Hodsoll, S. Ambwani, H. Himmerich, K. Rowlands, V. Cardi, J. Treasure

**Affiliations:** 1 Centre for Research in Eating and Weight Disorders (CREW), Department of Psychological Medicine, Institute of Psychiatry, Psychology and Neuroscience, King’s College London; 2 Department of Biostatistics and Health Informatics, Institute of Psychiatry, Psychology & Neuroscience, King’s College London, London, United Kingdom; 3 DIS Study Abroad in Scandinavia, Copenhagen, Denmark; 4 Bethlem Royal Hospital, South London and Maudsley NHS Foundation Trust, London, United Kingdom; 5 Department of General Psychology, University of Padova, Padova, Italy

## Abstract

**Introduction:**

Parents often remain the primary carers for their adult children with anorexia nervosa (AN). Carer burden and unhelpful behaviours have been extensively investigated as contributors to the maintenance of the illness and suggested several differences between fathers and mothers. However, the skilful dimensions of parental caregiving have been overlooked, and no longitudinal research has examined the caregiver abilities and psychological well-being of parents during their child’s transition from an intensive treatment.

**Objectives:**

This exploratory study compares the trajectories of change in perceived carer skills and psychological distress between fathers and mothers of adult patients with AN over 18 months following discharge.

**Methods:**

52 fathers and 224 mothers of adult AN patients were included in this study. A secondary analysis was conducted on data from the TRIANGLE trial using linear mixed-effects models. Outcomes were the total and subscale scores from the Caregiver Skills Scale (CASK) and the Depression, Anxiety, and Stress scale (DASS-21) in fathers and mothers at baseline, 6, 12, and 18 months.

**Results:**

Post-hoc tests revealed that fathers and mothers did not show significant differences in the CASK scores at any time-point (except for higher paternal “Self-Care” and “Biting-your-tongue” skills at baseline). However, the trajectories of change in their abilities differed between parents. Mothers showed significant increases over time in the overall CASK and in several of the subscales compared to fathers from baseline to month 18, but not months 6 or 12. In contrast, the changes in distress levels (DASS-21) followed similar trajectories for fathers and mothers over time.

**Conclusions:**

Fathers and mothers may have distinct needs in order to enhance their caregiving skills in the long-term. Although the findings require validation in future research involving father-mother dyads, effort should be made to ensure that carer support is tailored to be fit-for-purpose for both fathers and mothers.

**Trial registration** The TRIANGLE trial whose parental data was used in this study was pre‐registered on the ISRCTN registry (ISRCTN14644379).

**Disclosure of Interest:**

None Declared

